# The Need for a Motor Assessment Tool for Children with Autism—An Opinion Article

**DOI:** 10.3390/diagnostics13122095

**Published:** 2023-06-16

**Authors:** Hasan Wael Battah, Meir Lotan, Danny S. Moran

**Affiliations:** Department of Health System Management, Ariel University, Ariel 40700, Israel; meirlo@ariel.ac.il (M.L.); danielm@ariel.ac.il (D.S.M.)

**Keywords:** autism, motor development, diagnosis

## Abstract

There is a lot of evidence that early developmental therapy achieves impressive therapeutic results for those who require it. Therefore, developmental follow-up, which includes the process of monitoring the child’s development over time, makes it possible to identify possible developmental problems and treat them from a young age. This assumption is true in relation to all children with developmental difficulties but is mainly true in the context of children with a diagnosis of autism. However, despite the abundance of developmental scales for the neurotypical population, there are currently no valid scales for assessing motor function for children with autism. The current article focuses on the presentation of the motor delay, identified according to the literature, in many of the children with autism and requires the provision of professional and compatible treatment for these children. This motor delay and the lack of a motor assessment tool for children with autism raises the need for an adapted motor developmental assessment tool, which will produce measurable results, to enable the monitoring of the aforementioned disability and the receiving of tailored treatment from the physiotherapists who deal with the development of children with autism at an early age. The article reviews common existing assessment tools for use in assessing normal development in children with autism, presents the limitations and the challenges that arise when using these assessment tools with children on the autism spectrum and presents the need for a new developmental assessment tool that will be built and validated specifically for children with autism.

## 1. Introduction

### 1.1. Motor Development

Milestones in child development are divided into a large number of areas of development, such as motor development, sensory development, mental development and social-emotional development [1].

There are two main types of motor development, gross motor and fine motor. Fine motor skills describe the activity of the small muscles in the palm of the hand that are used to operate objects and function in daily activities, such as buttoning buttons, cutting, tying shoelaces and more. On the other hand, gross motor skills describe the activity of the large muscles of the body and include the postural mechanisms, the balance system and mobility in large activities of the body, such as walking, running and jumping [2].

Development in all areas is affected by the maturation of the child’s physiological and brain systems and learning from experience in the environment [1]. The rate of development of each child is different and, as long as the ability develops within the normative age range, it is normal. However, findings of delayed motor development and poor-quality movement performance may indicate the existence of motor problems, which require clinical intervention, in light of the fact that early detection, diagnosis and treatment (early intervention) contribute to promoting the child’s development, health and readiness for future tasks [3]. Delayed motor development and impaired performance are widely discussed in the literature among children with various medical diagnoses. One area that has been extensively discussed in the literature is that of children with autism spectrum disorder (ASD) [4].

### 1.2. Autism Spectrum Disorder (ASD) 

Autism is currently one of the most visible and widely discussed human conditions and its increased prevalence has brought it to the attention of society in the United States (U.S.), with world-wide recognition [5], and is one of the most common neurodevelopmental disorders [6]. The prevalence of ASD has been steadily increasing over the past two decades [7]. Approximately 1/100 children are diagnosed with ASD worldwide [8] and it is evident in all racial, ethnic and socioeconomic groups [9]. 

Autism is defined on the basis of social and communication problems and repetitive and restrictive behaviors that can vary in individuals along a continuum of severity [4]. A diagnosis of autism can be made as early as 18–24 months of age; it is around this age that characteristic symptoms can be distinguished from typical development and from other delays or other developmental conditions [8], while in the U.S. the average age of diagnosis is 50 months [10].

### 1.3. Autism Spectrum Disorder (ASD) Linked to Motor Development

The official diagnosis of autism does not include motor problems and in the *Diagnostic and Statistical Manual of Mental Disorders*, Fifth Edition (DSM-5), it is mentioned in only a single sentence that some of these children present motor difficulties. The familiarity with this population in the clinical setting suggests a considerable motor deficiency in most of them, and this observation is also supported by the scientific literature. The next part of the article presents the findings to date in the context of motor abilities and the motor development of children with a diagnosis of autism.

### 1.4. Late Motor Development and Challenges in Motor Performance in Autism

Motor disability among children with autism or Kanner’s Syndrome was described in a general way for the first time in 1943 by Kanner, as part of the definition of autism, Kanner noted that “motor disorders are often detected and include strange gait, clumsiness and other motor signs” [11]. Despite this initial report by Kanner, for years the academic literature ignored these motor impairments and sometimes even described the movement of the child with autism as smooth and coordinated. However, many studies during the last two decades have indicated that the achievement of motor milestones is delayed in children with autism and that there is an impairment in the quality of movement that is common among children with ASD [12,13]. 

In fact, a retrospective study of motor behavior in infants using video analysis found that the infant with ASD had significantly less static and dynamic symmetry in the supine position at 12–21 weeks [14]. Another study suggested asymmetrical movement patterns, such as Righting from Supine to Prone, sitting, crawling, walking [15] and delayed age of motor acquisition, e.g., sitting without support, standing without support [16], first walking [17] and walking alone [16].

In addition, the results of other research showed that motor development of infants with a high risk for ASD at age 6 months was correlated with ASD status at age 24–36 months, i.e., ASD was associated with lower early motor abilities [18]. Other research suggested that ASD children exhibited a developmental level similar to non-ASD children at age 6 months, but thereafter they showed atypical trajectories. Impairment from 14 to 24 months prevailed in the early-ASD compared to the later-ASD group, but was similar at 36 months [19].

In the past, prospective studies of at-risk infants have documented other atypical phenomena, ranging from gaps in age-appropriate motor abilities and gaps in early motor skills to more serious difficulties [20,21,22,23]. Furthermore, a recent prospective study evaluated the motor domain in infants with early autism signs aged 9–14 months. At baseline motor difficulties were very frequent, affecting the majority of the infants in the gross motor domain and fewer of the infants in the fine motor domain. At a 6-month follow-up, few of the infants maintained gross motor difficulties, while the majority of the infants continued to show fine motor difficulties [24]. 

Some researchers [25] even believe that motor impairment is actually the basis for the development of autism in children. In other words, an infant’s coordinated motor response is the basis for an initial response in the face of the initial communication actions of the other people in his life and, since the infant’s first motor response is impaired, this deficiency subsequently leads to impaired primary communication that is then the basis for the development of impaired communication patterns throughout the life of the adolescent and adult child with autism. In addition, one of the main developmental tasks of infancy is represented by exploration. The acquisition of new and more complex gross and fine motor abilities allows infants to obtain more information about their social and physical worlds. If these advances are slowed, this potential for exploration and learning opportunities decreases [4]. 

In addition, these researchers argue that motor learning is usually achieved through observation and imitation. Therefore, the original motor impairment, combined with a social difficulty that comes as a result of that original impairment, subsequently leads to emotional problems, up to the development of social behavior and poor communication that is finally diagnosed as the main impairment in those children with autism [25].

The backlog of these studies actually indicates that the motor disability exists at the base of the autism diagnosis and is in fact one of the core disabilities of the child, the adolescent and the person with autism, but despite these findings this disability is not fully treated in this population at any age. Children, adolescents, and adults with autism have been found to exhibit a variety of motor impairments in standard motor assessments that have been validated for children without disabilities. Children and adults with autism whose ages ranged from 7–32 years have been repeatedly found to have poor coordination during tasks requiring balance, ball play, agility and speed [26,27,28,29].

The findings regarding motor impairment in children with autism are currently based on a range of literature and indicate deficiencies in the following areas.

In some research investigations, impairments in gross as well as fine motor skills have been reported: gross motor skill impairments such as running, jumping [30], ball catching skills [31,32], ball throwing skills [30,31,32], ascending stairs and jumping upwards [33], balance skills such as one-board balance, walking heel-to-toe forwards, and hopping on mats [32]. Furthermore, the greatest deficits were in the object control domain, which includes skills related to manipulating and moving objects (e.g., throwing, catching, and kicking) while, reaching was found to be the least impaired gross motor skill [34]. Likewise, skills recruiting the lower extremities are less impaired than skills recruiting the upper extremities in ASD [34]. In addition, researchers have demonstrated that motor impairments are also common in gait patterns of children with ASD. They perform longer stance phases and shorter steps, using a greater base of support, greater hip flexion, less knee extension, and an altered contact pattern. Differences in gait cadence and hip and ankle kinetics have also been found, with reduced plantar flexor movements and increased dorsiflexion angle [35,36].

Fine motor skill impairments, such as in manual dexterity, difficulty holding and manipulating small objects, cutting with scissors, and performing a variety of dressing skills [37], were also reported in children with ASD. Other reports regarding fine motor challenges in this group of children included: hand function during ADL activities and writing skills [31], tracing [28], slowness in prehension movement and planning and execution [38], and other activities such as buttoning, zipping clothing, and assembling toys, which together can pose problems for children with ASD [39]. In addition, children with autism tend to have late onset of hand preference and differ qualitatively in their performance [39]. Early pioneers studying prehension have determined differences in dominant and non-dominant hand in children’s performance [40].

Other researchers have also reported impaired ability to imitate a sequence of motor movements, with these being found to be highly correlated with dyspraxia that manifests itself in slowness of movement, difficulty in planning and executing movement, problems with balance or poor coordination [41,42,43,44,45], such as motor impairments in board balance, balance coordination, balance ball/wall [31], and often low capacity for motor learning [27,46]. In addition, children with ASD have a more difficult time with non-object oriented imitation than they have with object oriented [47,48] and they were less successful with object oriented imitation than body movement orientation [45].

Moreover, children with ASD have greater difficulties in movement tasks that have an inherent dual nature, involving both accuracy and timing, as seen in the timed peg-board tasks and standing on one leg for as long as possible. This suggests that complexity of the motor task may be the important feature affecting performance [31].

Additionally, in the sensory field, the literature reports a sensory impairment on various levels [49] that impairs the child’s ability to cope with motor challenges. In other words, sensory feedback plays a crucial role in the creation and control of self-movement in the first place. For example, motor control relies to a large extent on sensory integration which is a process mediated by attention and requires order in cognitive processing, and therefore difficulty in motor planning reflects poor use of external feedbacks, such as visual cues. In addition, difficulties in performance, such as perseverance, poor planning, and inflexible thinking style, emphasize part of the atypical motor sequence in autism, especially difficulties in redesigning movements [24].

In addition to these, the literature also reports problems in postural control, such as position, sway or ankle control when standing, especially in situations where it is difficult to rely on the visual feedback on which children with autism tend to base their posture [48,50,51,52].

Moreover, they found that genetic risks for ASD might be related to delays in the gross motor domain, as well as in the receptive language [53]. In light of the genetic background that ASD syndromes have, it is estimated that the recurrence rate of ASD in siblings of children with a diagnosis of ASD is between 10–20% [54,55]. Therefore, several prospective studies are currently being conducted with babies who have an older brother with a diagnosis of ASD [56]. The ability to identify a risk group raises the importance of checking for early signs in that group to allow earlier identification and intervention for those who are most at risk [54,55].

In view of the motor difficulties presented by children with a diagnosis of autism, the clear need for motor assessment for this population arises. For this purpose, we will review the common assessment tools [57] and the most reliable [58], which are currently used for diagnosis among the neurotypical population, including the most psychometrically suitable motor assessment tools for children with ASD [59], despite the fact that these scales were not constructed for this population.

### 1.5. Common Assessment Tools for Motor Assessment in Children with Neurotypical Development

The most reliable assessments [58] and commonly used assessment tools for motor assessment in children with neurotypical development [57], which include the most psychometrically suitable motor assessment tools for children with ASD [59] of all the tools currently in use, are:

1. *Movement Assessment Battery for Children—(MABC-2)*
2. *Test of Gross Motor Development, Second Edition—(TGMD-2)*
3. *Peabody Developmental Motor Scales, Second Edition—(PDMS-2)*
4. *Bruininks-Oseretsky Test of Motor Proficiency, Second edition—(BOT-2)*

1. *Movement Assessment Battery for Children* (MABC-2):

The MABC-2, a revised version of the movement assessment battery for children MABC, is used to identify motor impairment and to provide a description of motor difficulties in children. It is also the test that is most frequently used by examiners to test the gross motor performance in children. There are two forms of the MABC-2 that comprise the performance test and the checklist. The performance test is designed to assess fine and gross motor skill movement difficulties in children aged 3 to 16 years. Conversely, the checklist is used by parents, caregivers or teachers to rate how a child manages everyday tasks encountered at home and in school. It requires 20–40 min to administer the performance test, while the checklist takes about 10 min to administer. Test materials and additional required equipment include chairs, a table and a clipboard [60]. Normative data were collected from a sample of 1172 children from the United Kingdom (U.K.) [58]. The tool was found to be reliable and valid [58].

2. *Bruininks-Oseretsky Test of Motor Proficiency*, Second Edition (BOT-2):

Currently in use is the version created in 2005. The tool was created due to the need to assess the skill of gross and fine motor development among those aged 4–21 years and is proposed as both a screening tool and a diagnostic tool for children who may have motor impairments. It is also used for student selection in school placement and as an evaluative measure of the effectiveness of an intervention in movement and functional skills performance [60]. Estimated time to perform the short test is 15–20 min and the full test requires 45–60 min [60]. Equipment and materials for administration of the assessment tool, such as a manual, easel, record form and exam booklet, are provided with the purchase of a kit [60]. Normative data were collected from a sample of 1520 children from the U.S. [58]. The tool is reliable and valid [58]. The BOT-2 assessment tool is the most psychometrically appropriate motor competency assessment for children with autism [59].

3. *Test of Gross Motor Development*, Second Edition (TGMD-2):

The most recent version of the tool was created in 2000. The test measures gross movement skills and gives a qualitative evaluation in children from 3 to 10 years old. It helps to identify those children who are significantly behind their peers in gross motor development and, after that, to plan improvement interventions [61]. Estimated time for execution is 15–20 min [58]. Required equipment for using this tool includes masking tape, chalk, traffic cones, 10–15 cm light ball, 20–25 cm playground ball, 15–20 cm sponge ball, tennis ball, 20–25 cm plastic or slightly deflated playground ball, and tape measure [62]. Normative data were collected from a sample of 1208 U.S. children [58]. The tool is reliable and valid [58] and is considered a valid criterion [52]. The TGMD-2 assessment tool is the second most psychometrically appropriate motor competency assessment for children with autism [59].

4. *Peabody Developmental Motor Scales*, Second Edition (PDMS-2):

Currently in use as an updated version created in 2000, the tool is designed to assess gross and fine motor skills for children from birth to 6 years [61]. It focuses on assessment and intervention or treatment programming for children with disabilities. The test manual states that the test estimates a child’s motor competence relative to his or her peers, determines the balanced development of fine and gross motor movement skills, identifies skill deficits and evaluates progress [62]. Therefore, it can be used as a research tool. Estimated time to take the assessment in this tool is approximately 45–60 min (20–30 for gross motor only) [58]. Required equipment for use of this tool is the test kit and additional required materials [62]. The tool was standardized on a sample of 2003 U.S. and Canadian children [58]. The tool was found to be reliable and valid and presents high psychometric values [58].

Table 1 provides a summary and overview and Table 2 shows an overview of the information of the normative data of all assessment tools that have been described previously.

## 2. Limitations of the Motor Assessment Scale for Use in Children with ASD

The limitation of these motor assessment tools for children with autism is that all of them excluded children with ASD in the normative sample, while they included neurotypical development. Therefore, the content of the scales is not suitable for the characteristics of children with autism. In addition, the TGMD-2 scale makes it possible to test gross motor skills only, while children with autism have delays and motor problems in fine motor skills too, so the use of this scale in the clinic is not comprehensive for children with autism. In addition, there is no short form in the PDMS-2, so the time required is too long for children with ASD and is not suitable for children above 6 years.

Moreover, due to the lack of adaptation for motor assessment of children with autism and the limitations that exist in relation to children with autism in all the assessments mentioned above, a very strong challenge arises when using these assessments among clinicians working with children with autism.

## 3. Challenges in Using the Assessment Tools Created for Neurotypical Population for Children with Autism

All the tools in the current review are the most reliable [58] and the most widely used for children who show normative development [57] and include the most psychometrically suitable motor assessment tools for children with ASD according to the current literature [59]; however, the assessment of children with autism with these tools is difficult and challenging [64], since these tools do not have validity for this population. In a study of about 500 physical therapists in relation to the use of motor assessments for children, it was found that the most frequent changes made by the physical therapists were with children with autism. These changes were made due to the duration of the assessment, lack of understanding of items by the child, impatience of the child, and poor level of the attention necessary to allow compliance with the standard requirements of the tool. The changes reported by the physical therapists in the above tools, when used in practice for children on the autism spectrum, included: providing demonstrations beyond what is required by the tool, providing feedback while performing contrary to the instructions of the tool, changing instructions, changing/adding body movements, adding the number of attempts of the child to perform the task and reducing the original requirements according to the guidelines applied to these tools [65].

The frequent use of the scores obtained through modified assessments conducted contrary to the guidelines of the creators of the tool violates the normative values of the assessment, and comparing the results for the motor performance of children with a delay relative to their peers would be erroneous [66,67]. Considering these changes, any treatment plan produced according to these findings will be inaccurate and unprofessional. A re-evaluation can also lead to completely different results compared to the initial evaluation. These changes, which have been reported frequently (in 90% of cases) by therapists in the clinical field, indicate the urgent need to diagnose the therapeutic needs of non-normative populations, such as children with autism.

Recent research indicated that the difficulties in motor skills frequently reported in this population are a significant and not very exploited clinical target for autistic people of all ages and should also be included in the DSM as part of the clinical characteristics of autism. Therefore, motor representation would signal the need to give focused attention to motor functioning in this population and would provide a clear framework for how motor differences fit into the broader diagnostic picture [68].

It is crucial to note that the poor motor ability detected in this population in turn leads to difficulty/inability, or discomfort, in retrying activities based on gross motor skills, where this lack of experience in turn may cause additional difficulties that will prevent children and adults with autism from engaging in physical activity [68], which may lead to a sedentary lifestyle with all the health implications associated with this, and of course increase the gap between the motor abilities of these children in their adulthood compared to their peers without a diagnosis of autism.

In light of these data, an adapted motor diagnosis that will enable the established detection of the motor problems in these children is highly important, with the aim of promoting tailored treatment, as well as counseling and guidance for normative development in children with autism, and treatment of motor development problems already seen in early childhood, so that they can be given a tailored therapeutic response that will advance their abilities.

Motor diagnosis and measurement of milestones in motor development are performed using valid and reliable assessment tools. To accurately detect the motor difficulties of each child it is important to use validated tools. However, studies from the clinical field indicate that there is a significant difficulty in using validated tools that are used in the normative population, when using these tools with children with a diagnosis of autism.

Another question that the reader should ask is: will the same early evaluation that would be carried out in an adapted tool, and which would lead to early and adapted treatment for this population, lead to a long-awaited change in the child being treated?

## 4. The Results of Motor Therapy in Children with Autism

In many studies it has been found that treatments of motor orientation in children with autism have a significant positive effect on various areas of the child’s life, such as potentially significant advances in the motor domain [69], decrease in body mass index [70], improvement in balance [71,72] and flexibility [71], reduction in self-stimulation [73], improvement in early social communication skills [4], improvement in academic abilities [74], improvement of sensory limitation [75], improvement in social, listening, turn-taking, and transition skills [76], reduced severity of symptoms [77], and improvement in sibling interactions; in fact, there are research data suggesting that early motor exploratory skills are associated with expressive vocabulary at age 1, 2, and 3.5 years, with cognitive skills in toddlerhood and childhood, and also with later academic skills [78].

These areas of development constitute core deficiencies defined in the diagnosis of autism. These positive findings from motor intervention with children on the autism spectrum raise the importance of specific motor diagnosis, which will allow early treatment of the motor problems presented by this population.

## 5. The Need for a Motor Assessment Tool Adapted for Children with Autism

Since there is no assessment tool for the development of motor milestones validated for children with autism, along with the challenges in the use of the tools mentioned above and the results of their use in children with autism, the need arises to build an adapted assessment tool.

This assessment tool is expected to have significant implications at various levels in the clinical field and in the research field. In the clinical field, this is both from the perspectives of the therapist and of the patient. From the point of view of the therapist, this assessment tool is expected to facilitate and make it possible to perform an appropriate test for children with autism, starting from an early age, according to the results of which it will be possible to detect challenges related to the difficulties in gross motor skills experienced by the child with autism and, as a result of this thorough diagnosis, to build a comprehensive and specific treatment plan accordingly. Such a tool will also make it possible to continuously monitor the child’s abilities and development during and following the treatment. From the patient’s point of view, assessment tools are expected to enable greater and more effective cooperation, without the need on the part of the therapist to make significant adjustments and changes in assessment tools (which in practice harm normative values and the validity of existing tools) and preventing the child from being monitored for years as he moves from therapist to therapist. In the field of research, an adapted tool assessment is expected to allow the researcher to test the effects of various motor interventions among children with autism, and allow comparisons to be made between different interventions, for example, which intervention program is more effective for children with autism, and, finally, it will be possible to draw reliable and correct results accordingly. This will allow researchers to make conclusions and give correct and professional advice to children with autism.

Moreover, an agreed recognition of the extent and relevance of motor difficulties in autism, such as in the DSM-6, will generate interest and increased funding for comprehensive intervention studies in this field. This kind of recognition will, in turn, also lead to early detection and evaluation in this population. Likewise, increased awareness of the high frequency with which autism and motor conditions such as developmental coordination disorders occur in parallel, can also provoke a combination of survey and motor assessment for each of the children diagnosed. In the future, it is even possible to consider developing short and readily available parent questionnaires that can help identify motor difficulties in children with autism [57] from a very young age.

## 6. Recommendation Points to Consider When Building a New Adapted Motor Assessment Scale for Children with Autism

Firstly, since it was found that more ASD symptoms correlate with impairmentin motor skills [79], the new scale should address different aspects of motor abilities, such as mobility, balance, agility, strength, muscle tone, and others.

Secondly, due to emotional issues in children with ASD [80], assessments should be performed by a person familiar to the child and in familiar situations.

Thirdly, in accordance with ecological principles, such a test should be performed by a trained professional team specializing in children with ASD, and in different daily environments in which the child is operating (home, playground, classroom, etc.).

Some children with ASD find it difficult to follow verbal commands, therefore the new scale should have the opportunity to add other contributing factors, such as singing, using pictures or multiple demonstrations.

As some children with ASD do not follow instructions, the scale should involve observational items enabling the assessment, with minor cooperation from the child.

Due to the poor quality of movement typical of so many with ASD, aside from numerical values, the new scale should have a space for remarks relating to quality of movement.

As dealing and treating children with ASD needs specialized therapists [81], implementing such a scale should be performed after relevant training.

## 7. Summary

Identifying children with developmental challenges in early childhood is crucial to enable appropriate, tailored therapeutic solutions that will optimize their abilities. Measuring motor developmental milestones for the neurotypical child population is performed using valid and reliable tools in order to accurately identify the motor difficulties of each child and to provide a therapeutic response tailored to specific needs from an early age. Many valid motor development assessment tools are currently in use, but none of these are validated for children on the autism spectrum. The lack of a valid motor assessment tools for children with autism has led to the use of normative development assessment tools, not validated for this group of clients. As for the above-mentioned predicaments, there is currently a significant difficulty in reporting the actual motor abilities of these individuals using the existing assessment tools. According to existing studies, this leads to using these scales in a non-orthodox manner, de-validating these scales, which may lead to unreliable results, incorrect clinical conclusions, difficulty in follow-up and an overall outcome of inaccurate and incorrect intervention plans for these children, in light of the fact that many children on the autistic spectrum have been found to have motor difficulties, and many studies show that various motor interventions with this population promote the core disabilities of the autism on top of motor advancement. Such achievements are academic abilities, social communication, reduced sensory issues, reduced stereotypical behavior, enhanced family connections, reduced severity of symptoms, etc.. In other words, motor intervention for children with ASD contributes to the improvement of motor abilities in particular, yet also supports advancements in other core issues of ASD. Consequently, due to the significant involvement of physical therapists in treating children with ASD, and due to improvement shown in the literature when intervening with this group of children and their motor skills, there is an urgent need to build an assessment tool adapted to the motor development of children with autism. An assessment tool adapted for children with autism is expected to give significant benefits to the therapist, the patient and the researcher.

In our opinion, this tool should be quick to use (up to 20 min), performed by a physical therapist who specializes in children with ASD and is familiar with these children, caregivers/parents might be present during the test, and with a one-step execution for each motor test, verbal and practical demonstration adapted to individual abilities of the child, and to the different levels of children with ASD, easily understood instructions for a child with ASD, all with the aim of creating a comprehensive, specifically adapted and professional method. The treatment plan should be accessible to these children from a young age and throughout their lives.

## 8. Conclusions

The current article highlights the fact that children with autism exhibit impairments and delays in motor skills. It also shows the gains in motor skills and other core aspects of ASD as a result of motor intervention. In order to provide a professional and adapted treatment plan, it is necessary to conduct a test using a valid scale for children with autism. As of today, and according to the latest literature, there are a large number of scales for evaluating motor abilities for children with neurotypical development, but none of them are adapted for children with autism. Using scales that are not adapted to children with autism may lead to incorrect results on which an intervention program that is not adapted correctly to the child might be built. Therefore, this study raises the need for an assessment tool for motor abilities, specifically adapted to children with autism, which will allow the therapist and the patient to perform the test in a better way, allow measurable results, an adapted, individualized treatment plan and the achievement of goals accordingly. An appropriate, specifically adapted motor assessment tool is expected to contribute significantly to the clinical and research fields.

## Figures and Tables

**Table 1:** Administrative aspects of the motor assessment tools.

Scale	Purpose	Domains Tested	Subscales Tested (Items)	Age (Y:M)	Assessment Time (min)	Equipment/Manual
MABC-2	Identifying and describing the movement difficulties of children and adolescents [60].	Gross motor, fine motor, balance [60]	Skills differ for each age band Manual Dexterity (3) Aiming & Catching (2) Balance Static (1) Balance Dynamic (2) [60]	3:0–16:0 [60]	the performance 20–40 the checklist about 10 [6]	Comprehensive manual/kit: £1191 Test kit provides most equipment [58]
TGMD-2	Identify those children who are significantly behind their peers and plan improvement intervention [61].	Gross motor [61]	Locomotor (6) Object Control (6) [61]	3:0–10:0 [61]	15–20 [58]	Kit includes manual and record form: £128 Equipment not included [58]
PDMS-2	Early detection of developmental delays. Assessment of motor competence identification of motor deficits and imbalances between the two motor domain. Establishment of individual intervention, monitorization of the individual development [63].	Gross motor, fine motor [63]	Reflexes (8) Stationary (30) Locomotion (89) Object Manipulation (24) Grasping (26) Visual-Motor Integration (72) [59]	birth—6:00 [61]	45–60 (20–30 for gross motor only) [58]	Comprehensive manual/kit: £553 Includes some but not all equipment required [58]
BOT-2	Assessment of fine manual control, manual coordination, body coordination, strength, and agility. Identify motor skill problems and/or specific motor deficits [61].	Gross motor, fine motor [58]	Fine Motor Precision (7) Fine Motor Integration (8) Manual Dexterity (5) Bilateral Coordination (7) Balance (9) Running Speed & Agility (5) Upper-Limb Coordination (7) Strength (5) [59]	4:0–21:0 [61]	FT: 45–60 ST: 15–20 [62]	Comprehensive manual/kit: £961 Test kit provides most equipment [58]

Y = Years, M = Month, Min = minute, FT = Full test, ST = Short test.

**Table 2:** Normative data.

Test	Year	Origin	N	Sample
MABC-2 [58]	2006	UK	1172	United Kingdom children
TGMD-2 [58]	1997–1998	USA	1208	American children
PDMS-2 [58]	1997–1998	USA and Canada	2003	American and Canadian children
BOT-2 [58]	2005	USA	1520	American children

UK = United Kingdom, USA = United state of America.

## Data Availability

The data presented in this study are available on request from the corresponding author.
